# Uncertainties Associated with Quantifying Climate Change Impacts on Human Health: A Case Study for Diarrhea

**DOI:** 10.1289/ehp.1002060

**Published:** 2010-10-06

**Authors:** Erik W. Kolstad, Kjell Arne Johansson

**Affiliations:** 1 Uni Bjerknes Centre, Bergen, Norway; 2 Bjerknes Centre for Climate Research, Bergen, Norway; 3 University of Bergen, Department of Public Health, Bergen, Norway; 4 University of Bergen, Centre for International Health, Research Group in Global Health: Ethics, Economics and Culture, Bergen, Norway

**Keywords:** climate change, diarrhea, future projections, global warming, human health

## Abstract

**Background:**

Climate change is expected to have large impacts on health at low latitudes where droughts and malnutrition, diarrhea, and malaria are projected to increase.

**Objectives:**

The main objective of this study was to indicate a method to assess a range of plausible health impacts of climate change while handling uncertainties in a unambiguous manner. We illustrate this method by quantifying the impacts of projected regional warming on diarrhea in this century.

**Methods:**

We combined a range of linear regression coefficients to compute projections of future climate change-induced increases in diarrhea using the results from five empirical studies and a 19-member climate model ensemble for which future greenhouse gas emissions were prescribed. Six geographical regions were analyzed.

**Results:**

The model ensemble projected temperature increases of up to 4°C over land in the tropics and subtropics by the end of this century. The associated mean projected increases of relative risk of diarrhea in the six study regions were 8–11% (with SDs of 3–5%) by 2010–2039 and 22–29% (SDs of 9–12%) by 2070–2099.

**Conclusions:**

Even our most conservative estimates indicate substantial impacts from climate change on the incidence of diarrhea. Nevertheless, our main conclusion is that large uncertainties are associated with future projections of diarrhea and climate change. We believe that these uncertainties can be attributed primarily to the sparsity of empirical climate–health data. Our results therefore highlight the need for empirical data in the cross section between climate and human health.

The global mean surface temperature has increased by 0.8°C in the past century and by 0.6°C in the past three decades (Hansen et al. 2006). Climate models also have projected further global warming by several degrees Celsius by the end of this century (Solomon et al. 2007), depending on future greenhouse gas (GHG) emissions. Climate change is expected to be an important health determinant for people in vulnerable areas (Confalonieri et al. 2007; Patz et al. 2005). There are concerns that climate change will cause excess deaths from malnutrition because of drought and crop failure (Schmidhuber and Tubiello 2007), diarrhea [McMichael et al. 2004; World Health Organization (WHO) 2004—referred to as WHO04 in this article], respiratory diseases (Beggs and Bambrick 2005), and vector-borne infectious diseases, such as malaria (Tanser et al. 2003).

When the impacts of climate change on health are estimated, uncertainties arise from many sources. In the words of Haines et al. (2006), “There is ... uncertainty over future climate change (particularly future greenhouse gas emissions), uncertainty about climate/health relations, and most importantly, uncertainties around the degree to which current climate/health relations will be modified by socioeconomic adaptation in the future.” These uncertainties must be presented in unambiguous ways in any study of the future health impacts of climate change (Campbell-Lendrum and Woodruff 2006). Otherwise, rather than being helpful for policymakers, such studies may give misleading projections of the impacts of climate change.

Here we have suggested a general approach for quantifying the impacts of future climate change on human health that incorporates uncertainties in empirical health data, as well as uncertainties associated with climate change projections. We have applied this approach to a specific case study of diarrhea incidence. The apparent tandem increase of diarrhea and temperature is one of the few reasonably well-studied statistical linkages between disease and climatic fluctuations.

To the best of our knowledge, WHO04 is the only existing quantitative study of the impacts of global warming on diarrhea. Using empirical studies from Fiji (Singh et al. 2001) and Peru (Checkley et al. 2000; Lama et al. 2004), WHO04 inferred that warming by 1°C was associated with a 5% increase in diarrhea, and noted that this was probably a conservative estimate. A wide uncertainty range (0–10% per 1°C warming) was applied to the correlation between diarrhea and temperature, but temperature projections from only one climate model were used. As discussed below, because of the substantial amount of intermodel discrepancy with respect to regional projections, it is common practice to use multimodel ensembles, that is, a set of results from multiple models, when assessing the spatial and temporal aspects of climate projections and forecasts (Collins 2007; Hagedorn et al. 2005; Tebaldi and Knutti 2007; Thomson et al. 2006).

In this article, we used the results from 19 state-of-the-art climate models to span the largest possible range of intermodel differences and the uncertainties of GHG radiative forcing. We also used recent empirical studies to narrow the uncertainty range associated with temperature–diarrhea regression coefficients in WHO04. Thus, our study is an extension of WHO04.

Currently, about 90% of all global deaths attributable to diarrhea occur in Africa, the Eastern Mediterranean region, and Southeast Asia (WHO 2008). Diarrhea has been estimated to account for 17% of all deaths among children < 5 years of age and is ranked as the fifth leading cause of death in the world (WHO 2008). Because of the complexity of the causal patterns of the deadliest diseases and the regional nature of climate change, evidence of direct links between disease and climatic parameters is scarce (Patz 2002). That said, there are persuasive reasons to fear that the prevalence of diarrhea will increase with climate change. For instance, temperature increases were found to be positively correlated with *Salmonella* in a number of European countries and Australian cities (D’Souza et al. 2004; Kovats et al. 2004) and with *Salmonella, Campylobacter*, and *Escherichia coli* in Massachusetts and at different sites in Canada (Fleury et al. 2006; Naumova et al. 2007).

The estimate of temperature–diarrhea correlation used in WHO04 is supported by a study from Dhaka, Bangladesh, where weekly diarrhea cases that were not related to cholera increased by 6% per 1°C increase (Hashizume et al. 2007). However, regional differences and contrasting effects of temperatures on different kinds of diarrhea are evident. For example, a recent study from Japan found that the weekly number of infectious gastroenteritis cases increased by 8% for every 1°C increase in the average temperature (Onozuka et al. 2010). In another recent study from two sites in China, Zhang et al. (2007) used regression analysis and found increases of 11–16% in the number of cases of bacillary dysentery for each 1°C temperature increase. Rotavirus, the leading cause of severe diarrhea globally, peaks in winter in temperate regions, but a 1990 meta-analysis found a less-distinct seasonality in the tropics (Cook et al. 1990). In another more recent meta-analysis, Levy et al. (2008) found a negative correlation between temperature and rotavirus in the tropics but with large differences between the sites. In the Dhaka study, Hashizume et al. (2007) found that the estimated effect of rising temperatures increased slightly when rotavirus cases were excluded. In a newer study from Dhaka, Hashizume et al. (2008) found that the number of rotavirus cases had a U-shaped distribution with respect to temperature, which indicated nonlinear effects at play that could not have been identified with linear regression models. Negative correlations between rotavirus diarrhea and temperatures were found up to a threshold of 29°C, but over this threshold, a 20–60% increase in hospital visits for rotavirus diarrhea was found with each 1°C temperature increase (Hashizume et al. 2008). We examined a range of quantitative associations between increases in diarrhea and temperature to reflect the range of uncertainty of the climate data, the range of regional variations, and the range of empirical correlations found between temperature fluctuations and different types of diarrhea.

Besides temperature, climatic factors such as rainfall, relative humidity, and air pressure may contribute to changes in incidence of diarrhea. The extent of these influences is highly dependent on the pathogens and on the water and sanitation infrastructure in different regions. The exact causal mechanisms are unclear, but all of these variables may have an impact on the replication rate of certain bacterial and protozoan pathogens (to different extents). They may also have an impact on the survival rates of different viruses. Furthermore, heavy rainfall may contaminate drinking water on a larger scale. Few papers have examined the relationships between other climatic factors and diarrhea, and the results are inconsistent. Some studies found that rainfall does not affect transmission of specific diarrheal pathogens (Zhang et al. 2007), whereas other studies found that low levels of rainfall are associated with high incidences of diarrhea (Singh et al. 2001). Therefore, it seems reasonable to choose to explore the uncertainties with the single factor already known to be correlated with diarrhea, rather than assessing the uncertainties associated with factors for which the links to diarrhea are less clear.

As we mentioned, uncertainties associated with the temperature response to increased radiative forcing due to GHG emissions are another important factor when projecting the impacts of climate change on human health. Although all climate models indicate that increases in radiative forcing from higher GHG concentration levels lead to global warming, they vary in their regional projections (Räisänen 2001). Even a single climate model may yield different responses if key model parameterizations are changed (Murphy et al. 2007). As a result, the future projections in the 2007 Fourth Assessment Report of the Intergovernmental Panel on Climate Change (IPCC) were based on data from 25 climate models, including the 25 used here. Each climate model was run multiple times for a common set of experiments, and each run was forced with preset pathways of greenhouse gas emissions, as specified by the IPCC’s Special Report on Emissions Scenarios (SRES; IPCC 2000).

We analyzed projected temperature changes in the regions that are currently most affected by diarrhea to highlight the large uncertainties that are associated with attempts to quantify the impact of climate change on human health. These uncertainties stem from the empirical data that relate climate change to health impacts and from the future projections of the climate models. By using an analysis of projected changes to the incidence of diarrhea as a case study, we have illustrated the importance of obtaining more robust empirical evidence of the causal correlations between relevant health outcomes and climate change. Our research highlights how today’s scarcity of empirical studies of climate change impacts health and illustrates how the disagreement between climate models act together to limit the precision of future projections. Our analysis indicates that simplistic models of the relationships between health and climate change may be misleading and may therefore give a false impression of the true health impacts of global warming.

## Materials and Methods

Data from 19 coupled atmosphere–ocean climate models from the World Climate Research Programme Coupled Model Intercomparison Project Phase 3 (CMIP3) multimodel data set (Meehl et al. 2007) were used to form a large multimodel ensemble. These models are listed in Table 1, along with the research and modeling groups that made the model data available. The future scenario projections in CMIP3 were implemented by each group in two steps. First, the observed radiative forcing, including past GHG concentrations and volcanic eruptions, was used as input in the models for the period 1850–2000. These model runs are known as the 20C3M simulations. The second step was to resume the model runs beginning in 2000 by imposing projected GHG emissions according to preset scenarios from the IPCC SRES (IPCC 2000).

In this study, we analyzed annual temperature projections for the SRES A1B emissions scenario (IPCC 2000), where future emissions are specified to increase rapidly beginning in 2000 and then gradually decrease until the atmospheric GHG concentration stabilizes at 720 ppm of CO_2_ equivalents toward the end of the 21st century. Two-meter temperatures were used, the standard level at which temperatures are measured at meteorological stations. The projected temperature changes are always presented as simulated changes relative to the simulated temperatures of the individual models for the baseline period 1961–1990. It is important to emphasize that many models have regional and even global temperature biases with respect to observations. These biases are not necessarily caused by errors in the models; many of the factors that determine the level of natural variability in the climate system appear to be nondeterministic (e.g., Lorenz 1976) and are not implemented in today’s climate models. It is therefore customary to compute the simulated changes in a given parameter with respect to a baseline period (under the assumption that the model biases are constant with global warming). We computed the 1961–1990 baseline temperatures from the 20C3M scenario simulations of the individual models, which were driven by observed 20th-century radiative forcing (GHG concentrations and volcanoes).

We defined the number α as the estimated percentage increase in the relative risk (RR) of diarrhea with each 1°C temperature increase. Thus, if Δ*T* is the projected temperature increase relative to 1961–1990 (in degrees Celsius), the RR after a temperature rise is RR = 1 + α × Δ*T*. The assumption that the association between diarrhea and a one-unit increase in temperature is constant across all possible temperature increases is at odds with the study from Bangladesh (Hashizume et al. 2008), where the increase in the RR of diarrhea with a 1°C increase in temperature was greater when the temperature exceeded a certain threshold. Similarly, Checkley et al. (2000) found that admissions due to diarrhea doubled during the 1997–1998 El Niño event, when the temperatures in Lima were up to 5°C above normal. These studies suggest that the relationship with temperature can be quite complicated for some diarrheal diseases. However, following the majority of the currently available studies, for the purposes of our study, we assume that α is constant with Δ*T*.

WHO04 used two studies, with α-values of 0.03 (Singh et al. 2001) and 0.08 (Checkley et al. 2000), to obtain an α-value of 0.05. We found five empirical studies, including those two conducted by Singh et al. (2001) and Checkley et al. (2000), that used linear regression models to isolate the effects of temperature on diarrhea in general, and these studies are listed in Table 2. Unfortunately, we do not know which of these values is the most realistic.

To quantify the range of uncertainties associated with the choice of α and the range of temperature projections, we used a simple approach. For each year and location, there are 19 temperature projections, and we used five values of α. We chose to weight all the models and α-values equally. By combining these values in the formula above, we obtained a two-dimensional matrix of RR projections with 95 (19 × 5) elements for each year and location. We refer to these matrices as RR projection matrices in the remainder of the paper. Next, we show empirical cumulative distribution functions (ECDFs) based on the projection matrices. By spanning all available values for both α and Δ*T*, these ECDFs give realistic estimates of a range of RR projections, as well as their associated uncertainties. The WHO04 used only one temperature projection (from one climate model) for each study region; thus, our study has placed more emphasis on intermodel ranges of climate uncertainty. In addition, because we did not choose one value for α, we explicitly show the two-dimensional uncertainties associated with diarrhea–temperature correlations.

## Results

**Figure 1:**
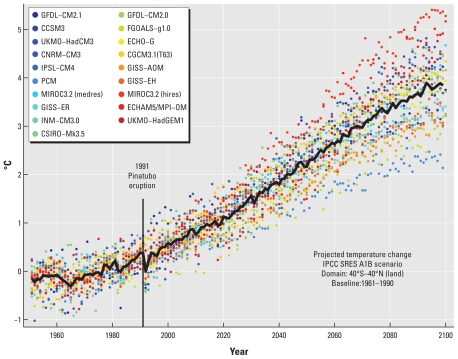
Temporal temperature projections for the tropics and subtropics. The black curve shows the ensemble average temperature from the 19 climate models under the A1B scenario, area averaged from 40°S to 40°N, and shown as annual changes with respect to the ensemble mean in the period 1961–1990. The colored dots show annual changes estimated by the individual models (see Table 1).

The projected annual mean temperature changes in the A1B scenario, with respect to the period 1961–1990 are shown in Figure 1. As the global impact of diarrhea is mainly confined to the tropics and subtropics, the data were area averaged for all dry (nonoceanic) grid cells (the locations for which the model computations are done, typically separated by a distance of 2–3 degrees) between 40°N and 40°S. The rationale for excluding oceanic grid cells is that the ocean surface does not warm as fast as the continents, so that the inclusion of wet grid cells would have introduced biases in our regional averages. The black curve shows the projected ensemble mean temperature evolution, and the colored dots show the annual mean temperature changes as simulated by the individual climate models. The models included anthropogenic GHG emissions, volcanic eruptions, and other observed radiative forcing up to the end of the 20th century. The major eruption of Mount Pinatubo in 1991 had a large impact on the global temperature (Soden et al. 2002), and the less powerful eruptions of Agung in 1963 and El Chichón in 1982 can also be discerned. As there were no major volcanic events in the models after 2000, the ensemble mean temperature fluctuations about the warming trend in that period are a result of interannual variability across the models and should be considered noise. The most striking feature in Figure 1 is the strong warming due to increased GHG concentrations after 2000. By the end of this century, the mean projected warming in the A1B scenario amounts to almost 4°C with respect to 1961–1990. Even the most conservative model shows a warming of more than 2°C by 2100. It is also worth noting that the level of discrepancy between the models increases as the temperatures rise.

**Figure 2:**
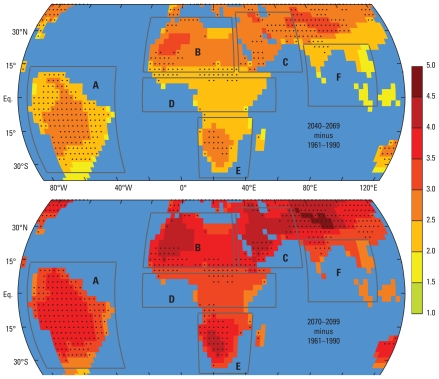
Spatial temperature projections for the tropics and subtropics. Temperature projections under the IPCC’s A1B scenario and with respect to 1961–1990 are shown for two time slices: 2040–2069 in the upper panel and 2070–2099 in the lower panel. Nineteen climate models were used, and the data were interpolated on a common grid. The unit is degrees Celsius, and the black dots show the grid cells for which the intermodel SD is higher than 0.5°C (top panel) and 0.7°C (bottom panel). The boundaries of regions are shown in dark gray: A, South America; B, North Africa; C, Middle East; D, equatorial Africa; E, southern Africa; F, Southeast Asia.

Figure 2 shows a map of the 19-model ensemble average warming of the tropical and subtropical landmass under the A1B scenario by 2040–2069 and 2070–2099, again with respect to the baseline period 1961–1990 in the 20C3M scenario. The data were interpolated on a regular global grid with 73 latitude and 144 longitude points globally, as the spatial resolution between the models varied. As discussed above, because warming of the oceans is slower than warming over land, the values in the dry grid cells on the target grid were composed using only dry grid cells from the source grids when interpolating. The projected temperature changes for the oceanic grid cells are not shown in Figure 2 because they are irrelevant in the context of this study.

The arid subtropical high-pressure belts, centered near 30°N and 30°S and spanning northern and southern Africa, the Middle East, and parts of Central Asia, are most susceptible to a strong warming across the range of models. Closer to the more humid equatorial regions in Africa, India, South America, and Southeast Asia, the warming is less pronounced because some of the GHG radiative forcing is used for evaporating soil moisture (Giannini et al. 2008). The black dots in Figure 2 indicate where the intermodel standard deviation (SD) of the warming exceeds the thresholds of 0.5°C (for 2040–2069, top panel) and 0.7°C (for 2070–2099, bottom panel). These thresholds were chosen somewhat arbitrarily to illustrate where the models are in disagreement. As indicated in Figure 1, there is some discrepancy between the models, and one of the highest levels of intermodel disagreement on the temperature change is found in South America.

**Figure 3:**
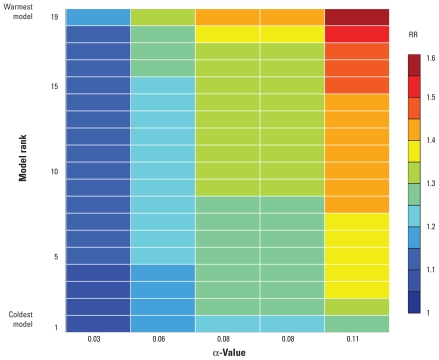
An example RR projection matrix. The projected changes to the RR of diarrhea, with respect to the 1961–1990 baseline, are shown for region B (North Africa as shown in Figure 2) for the period 2070–2099. The *x*-axis shows the five empirically derived increases in the RR of diarrhea for each 1°C temperature increase (α), and along the *y*-axis the 19 climate models are sorted with respect to the magnitudes of their projected warming.

We defined six geographical regions: South America, North Africa, Middle East, equatorial Africa, southern Africa, Southeast Asia (Figure 2) and computed 18 matrices of RR projections (one for each region, during 2010–2039–2040–2069, and 2070–2099) based on the 19 climate model temperature projections and five α-values. To illustrate this, an example of such a matrix is shown in Figure 3. We averaged the temperature projections in time over the period 2070–2099 (Δ*T* was computed with respect to 1961–1990) and the area over region B (North Africa) in Figure 2. One important feature in the figure is that the variance is larger across the columns than across the rows. This result implies that the estimates used to quantify the effect of temperature on diarrhea (α) have a larger impact on the uncertainties in RR projections than do the intermodel temperature projection variance (Figure 3). This aspect is discussed below.

**Figure 4:**
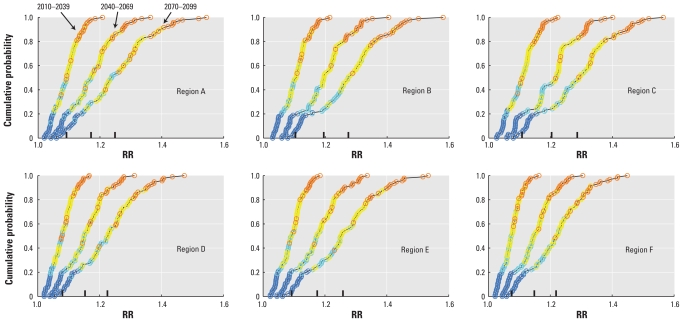
Projected changes of diarrhea with climate change. The projected changes to the RR of diarrhea, with respect to the 1961–1990 baseline, are shown as empirical cumulative distribution functions (ECDFs) for regions A–F. In each plot, all the values in the RR projection matrices are shown for three time periods: 2010–2039 to the left, 2040–2069 in the middle, and 2070–2099 to the right. The values are shown with distinct colors according to the corresponding α-values, that is, the empirically derived increases in the RR for each 1°C temperature increase. Blue colors correspond to α = 0.03, turquoise to α = 0.06, yellow to α = 0.08 and orange to α = 0.11.

After dividing the period 2010–2099 into three 30-year periods of equal length, we computed matrices corresponding to the one in Figure 3 for all six regions indicated in Figure 2. The ECDFs based on these matrices are shown in Figure 4. For each α-value, a distinct color code was used. The mean values for each matrix are shown on the *x*-axis. These values are also listed in Table 3, along with the SDs for each projection matrix. The WHO04 projections for developing countries suggested an 8–9% increase in the RR of diarrhea by 2030 compared with 1961–1990. These values are consistent with our mean projected increases of 8–11% for the period 2010–2039 (Table 3). Later in this century, for the periods 2040–2069 and 2070–2099, the mean of our projection matrices gives risk increases of 15–20% and 22–29%, respectively.

## Discussion

As empirical data are scarce and the nature of the relationship between climate and health is easily obscured by a large number of confounding factors, it is extremely important to treat the uncertainties associated with climate change impacts in a transparent manner. To illustrate this issue, we attempted to quantify the impacts of projected regional warming on diarrhea using the following tools: a range of linear regression coefficients α to express the relationship between temperature and diarrhea incidence, and 19 climate models in which the atmospheric concentration of GHGs was specified to increase according to an emissions scenario specified by the IPCC.

We found that the choice of α had a greater influence on the uncertainties associated with projected RRs of diarrhea than the choice of climate model, although the influence of the climate models increased with larger projected temperature changes toward the end of the century. Figure 4 and Table 3 illustrate that picking one α-value and using the temperature projections of only one climate model can be misleading. But perhaps the most important insight to be gained from Figure 4 and Table 3 is that the part of the variance that originates from intermodel discrepancies is relatively small if one ignores the outliers among the models, that is, the very coldest and warmest model projections. This result is a strong argument for using more than one model but also highlights the fact that the total variance of the RR projections is dominated by the width of the range of α-values. To further highlight this important result, Supplemental Material, Figure 1 (doi:10.1289/ehp.1002060) shows the range of projections for each region and time period when α was held fixed as the mean of the five values (while Δ*T* varied as before) in red, and when Δ*T* was held fixed as the intermodel mean (while α varied) in blue. In all cases, the range of RR projections is larger when the full range of α-values is used than when the intermodel range of Δ*T* is used.

The assumption that a universal value exists for α is clearly open to question and has been put in doubt by one study (Hashizume et al. 2008), where a distinctly nonlinear increase in rotavirus diarrhea above a certain temperature threshold is suggested. Nonlinear increases in diarrhea were also observed in Peru during the 1997–1998 El Niño episode, in which the temperature soared to up to 5°C above normal. This indicates that it might be more appropriate to study impacts from changes to the extreme values of daily mean temperatures, (rather than impacts from changes to annual mean temperature as in this study) for investigating diarrhea–temperature relationships, but we found no empirical data built upon this relationship. Therefore, an obvious and important item for future work is to develop nonlinear regression models for temperature impacts on diarrhea; however, this work requires more accurate empirical data. An improvement in this field might lead to decreases in the uncertainties of long-term projections of diarrhea and might also raise the prospect of seasonal forecasting of diarrhea, as has been done for malaria (Thomson et al. 2006).

Our study estimates some of the potential effects of projected climate change on diarrhea by using empirically derived estimates of the relationship between temperature and diarrhea incidence. Ideally, one could have performed stratified analyses for different pathogens, transmission routes, and disease severities, but this was impossible with the limited information offered in the studies that we used. Such an analysis is also beyond the scope of our study, which can be considered a high-level meta-analysis of the relationships between diarrhea in general and global warming.

The discrepancies among the studies with regard to α (Table 2) may be attributable to a range of biases and may also be caused by variations in the epidemiologic characteristics of the different population subgroups. It is not for us to rigidly assess the realism of one study over another. Nevertheless, we see from Table 2 that the two studies with the highest and lowest α-values have some characteristics that may cast doubt on their compatibility with the other studies. In the study with an α*-*value of 0.03 (Singh et al. 2001), monthly diarrheal admission reports were used in the regression analysis, whereas the other studies used daily or weekly reports. The study with an α*-*value of 0.11 (Lama et al. 2004) considered only an adult population; all the other studies included younger age groups in their analysis. These differences highlight the need for consistent and specific kinds of empirical data on the association between temperature and diarrhea. Future research to address existing information gaps should carefully consider the most appropriate case definitions and populations to evaluate for this purpose.

The uncertainties associated with regional climate projections are currently too large to be ignored. This is due partly to the coarse horizontal resolution of today’s climate models. In 2010 and 2011, the largest climate model data set so far, CMIP5, will be finalized and made ready for use in the scheduled IPCC Fifth Assessment Report. In contrast to earlier versions of CMIP, CMIP5 will have a substantial focus on regional scenarios and near-term decadal predictions (Taylor et al. 2009). We hope and expect that this joint modeling effort will yield improved future regional temperature projections. However, for more accurate scenarios in densely populated areas, very high-resolution simulations will still be needed. For instance, urban heat island environments (Arnfield 2003) can be substantially warmer than their surroundings. To obtain realistic temperature projections for such locations, accurate descriptions of important factors such as land use and topography must be fed into very-high-resolution regional climate models (Giorgi and Mearns 1991).

We emphasize that the possible effects of future adaptive health policies on diarrhea prevalence have not been assessed in this study. A recent study estimated that a 40% reduction in baseline rotavirus mortality could be achieved through mass vaccinations (Rose et al. 2009). In a recent international policy plan that aims to reduce childhood deaths from diarrhea, the United Nations Children’s Fund (UNICEF) and the WHO presented a comprehensive seven-point plan for diarrhea control (WHO, UNICEF 2009). This policy document does not assess in a quantitative manner the impacts of climate change and policy adaptations on the global burden of diarrhea. Nevertheless, investments in sanitation and access to safe drinking water, together with increased access to vaccines, will obviously lessen the projected increase in the burden of diarrhea induced by global warming.

Our results show that future climate change may bring disastrous increases in diarrhea. However, our most important result is that the uncertainties associated with these increases are unacceptably large. More accurate empirical data for the relationships between climate and health are clearly needed, as highlighted by Campbell-Lendrum and Woodruff (2006). We echo the call for action by Huntingford et al. (2007):

Currently we see a gulf between the climate modelling research community, which provides predictions of future ‘weather’ such as temperature, humidity and rainfall, and those in the medical research community, which studies epidemics and the incidence of disease. Yet there is a crucial need to warn the policy- and decision-makers faced with the need to implement adaptive strategies of the future human health impacts of ‘global warming.’ To fulfill this need, climate and medical scientists must work together.

## Figures and Tables

**Table 1:** The 19 climate models and their corresponding institutions.

Country	Originating group(s)	CMIP3 model(s)
Australia	CSIRO Marine and Atmospheric Research	CSIRO-Mk3.5
Canada	Canadian Centre for Climate Modelling and Analysis	CGCM3.1(T63)
China	LASG/Institute of Atmospheric Physics	FGOALS-g1.0
France	Météo-France/Centre National de Recherche Météorologiques (CNRM)	CNRM-CM3
France	Institut Pierre Simon Laplace	IPSL-CM4
Germany	Max Planck Institute for Meteorology	ECHAM5/MPI-OM
Germany/Korea	Meteorological Institute of the University of Bonn, Meteorological Research Institute of Korean Meteoological Adminstration, and Model and Data Group	ECHO-G
Japan	Center for Climate System Research (University of Tokyo), National Institute for Environmental Studies, and Frontier Research Center for Global Change (JAMSTEC)	MIROC3.2 (hires) and MIROC3.2 (medres)
Russia	Institute for Numerical Mathematics (INM)	INM-CM3.0
United Kingdom	Hadley Centre for Climate Prediction and Research/Met Office (MO)	UKMO-HadCM3 and UKMO-HadGEM1
United States	National Center for Atmospheric Research	CCSM3 and PCM
United States	U.S. Department of Commerce/ National Oceanic and Atmospheric Administration /Geophysical Fluid Dynamics Laboratory (GFDL)	GFDL-CM2.0 and GFDL-CM2.1
United States	National Aeronautics and Space Administration/Goddard Institute for Space Studies (GISS)	GISS-AOM, GISS-EH, and GISS-ER

Abbreviations: AOM, Atmosphere–Ocean Model; CCSM3, Community Climate System Model Version 3; CGCM3.1, Third Generation Coupled Global Climate Model; CM, Climate Model; CSIRO, Commonwealth Scientific and Industrial Research Organisation; ECHAM5/MPI-OM, Max Planck Institute for Meteorology Atmosphere and Ocean Model; ECHO-G, Hamburg Atmosphere–Ocean Coupled Circulation Model; EH, ModelE20/HYCOM 4×5×L20 ; ER, ModelE20/Russell 4×5×L20; HadCM3, Hadley Centre Climate Model Version 3; HadGEM1, Hadley Centre Global Environmental Model, version 1; hires, high resolution; LASG, National Key Laboratory of Numerical Modeling for Atmospheric Sciences and Geophysical Fluid Dynamics; medres, medium resolution; MIROC, Model for Interdisciplinary Research on Climate; PCM, Parallel Climate Model.

**Table 2:** A summary of the five empirical studies used to determine the increase in the RR of diarrhea for each 1°C temperature increase (α).

Study	Region	Estimated α (95% CI)	No. of participants	Outcome measure	Population	Period
Checkley et al. 2000	Lima, Peru	0.08 (0.07–0.09)	57,331 admissions due to diarrhea	Daily admissions of diarrhea cases at one diarrheal unit in one hospital	Children < 10 years of age	1993–1996
Singh et al. 2001	Fiji	0.03 (0.01–0.05)	Not available	Regional database of monthly diarrhea cases based on reports from multiple hospitals	All age groups	1978–1989
Lama et al. 2004	Lima, Peru	0.11 (0.07–0.16)	237,382 admissions (40,020 due to diarrhea)	Monthly admissions of diarrhea cases at the emergency unit in one hospital	Adults > 13 years of age	1991–1998
Hashizume et al. 2007	Dhaka, Bangladesh	0.06 (0.03–0.08)	12,182 admissions due to diarrhea	Weekly admissions of noncholera diarrhea cases at one diarrheal unit in one hospital	All age groups	1996–2002
Onozuka et al. 2010	Japan	0.08 (0.05–0.11)	422,176 reported cases of infectious gastroenteritis	Regional database of weekly infectious gastroenteritis cases (defined as sudden stomach ache, vomiting, and diarrhea) based on reports from multiple hospitals	All age groups	1999–2007

CI, confidence interval.

**Table 3:** Projected RR (SD) of diarrhea in the 21st century relative to the baseline period 1961–1990.

	Time period		
Region	2010–2039	2040–2069	2070–2099
A–South America	1.09 (0.04)	1.17 (0.07)	1.25 (0.11)
B–North Africa	1.10 (0.04)	1.19 (0.08)	1.27 (0.11)
C–Middle East	1.11 (0.05)	1.20 (0.08)	1.29 (0.12)
D–Equatorial Africa	1.08 (0.04)	1.15 (0.06)	1.23 (0.10)
E–Southern Africa	1.09 (0.04)	1.18 (0.07)	1.26 (0.11)
F–Southeast Asia	1.08 (0.03)	1.15 (0.06)	1.22 (0.09)

For each time period and each region, data is listed as the mean of the RR projection matrix.
